# Estimates of genetic parameters for fat yield in Murrah buffaloes

**DOI:** 10.14202/vetworld.2016.295-298

**Published:** 2016-03-19

**Authors:** Manoj Kumar, Vikas Vohra, Poonam Ratwan, Jamuna Valsalan, C. S. Patil, A. K. Chakravarty

**Affiliations:** 1 Division of Dairy Cattle Breeding, ICAR-National Dairy Research Institute, Karnal - 132 001, Haryana, India; 2 Department of Animal Genetic Resources, ICAR-National Bureau of Animal Genetic Resources, Karnal - 132 001, Haryana, India; 3 Department of Animal Genetics and Breeding, Lala Lajpat Rai University of Veterinary & Animal Sciences, Hisar, Haryana, India

**Keywords:** genetic factors, Murrah buffalo, non-genetic factors, test-day fat yields

## Abstract

**Aim:**

The present study was performed to investigate the effect of genetic and non-genetic factors affecting milk fat yield and to estimate genetic parameters of monthly test day fat yields (MTDFY) and lactation 305-day fat yield (L305FY) in Murrah buffaloes.

**Materials and Methods:**

The data on total of 10381 MTDFY records comprising the first four lactations of 470 Murrah buffaloes calved from 1993 to 2014 were assessed. These buffaloes were sired by 75 bulls maintained in an organized farm at ICAR-National Dairy Research Institute, Karnal. Least squares maximum likelihood program was used to estimate genetic and non-genetic parameters. Heritability estimates were obtained using paternal half-sib correlation method. Genetic and phenotypic correlations among MTDFY, and 305-day fat yield were calculated from the analysis of variance and covariance matrix among sire groups.

**Results:**

The overall least squares mean of L305FY was found to be 175.74±4.12 kg. The least squares mean of overall MTDFY ranged from 3.33±0.14 kg (TD-11) to 7.06±0.17 kg (TD-3). The h^2^ estimate of L305FY was found to be 0.33±0.16 in this study. The estimates of phenotypic and genetic correlations between 305-day fat yield and different MTDFY ranged from 0.32 to 0.48 and 0.51 to 0.99, respectively.

**Conclusions:**

In this study, all the genetic and non-genetic factors except age at the first calving group, significantly affected the traits under study. The estimates of phenotypic and genetic correlations of MTDFY with 305-day fat yield was generally higher in the MTDFY-5 of lactation suggesting that this TD yields could be used as the selection criteria for early evaluation and selection of Murrah buffaloes.

## Introduction

India has about 51 million milch buffaloes [1] contributing about 51% [2] of the total milk produced in the country. Compared with cow’s milk, buffalo’s milk has a higher percentage of fat percentage. The reported values of fat percentage for buffalo’s milk varies from 6.87% to 8.59% [3,4]. In spite of its higher fat percentage, milk cholesterol content is lower in buffalo’s milk compared to cow’s milk, which is 275 mg versus 330 mg as reported by Zicarelli [5]. Milk fat plays a significant role in the nutritive value and physical properties of milk and milk products. Besides serving as a rich source of energy, fat contains significant amounts of essential fatty acids-linolenic and arachidonic acid. The most distinctive role which milk fat plays in dairy products concerns flavor. Nowadays, milk pricing system is also based on the percentage of fat in milk, therefore, higher milk fat yield fetches better economic returns. Murrah is the most important buffalo breed with superior genetic potential for milk fat yield production.

To find out an alternative to daily milk yield recording, which is a costly and time-consuming proposition under field conditions, some studies have been made in the past in buffaloes on test day (TD) milk yields [6,7]. Various advantages of using TD milk yield records are individual test date effects, and the number of records per animal as well as the interval between records can be accounted for better adjustment of non-genetic factors influencing the milk yield leading to more accurate genetic evaluation. Today, in many countries across the continents, multi trait evaluations are employed in genetic evaluations. TD milk fat yield records can be used in combination for more accurate genetic evaluation. Although TD fat yield records offer greater advantage compared to 305-day fat yield in selection schemes, information on estimation of genetic parameters based on TD records particularly, monthly records are limited.

The present investigation was undertaken with the objective to study the influence of various non-genetic and genetic factors on monthly TD and lactation 305-day fat yields (L305FY) and to estimate the genetic parameters for milk fat yield, which could be used for selecting Murrah buffaloes for higher fat yield.

## Materials and Methods

### Ethical approval

The experiment was conducted following the code of ethics for animal experimentation with approval from the Institute’s Animal Ethics Committee.

### Data

A total of 10381 monthly TD fat yield (MTDFY) records comprised first four lactations of 470 Murrah buffaloes calved during 1993-2014 at the ICAR-National Dairy Research Institute, Karnal were collected from the history-cum-pedigree sheets and monthly record of milk yield and fat percentage register. The traits considered for analysis were MTDFY and L305FY. Culling in the middle of lactation, abortion, stillbirth, or any other pathological causes affecting the lactation yield were considered as abnormalities and thus, such records were not taken for the study. Records of buffaloes with <500 kg of milk production and covered <100 days of lactation, were not considered, a set practice at our herd, as usually such animals had shown good average daily milk yield. To ensure the normal distribution, the outliers (µ±3 standard deviation) were removed, and data set was standardized. The data were analyzed to study the effect of non-genetic factors (parity, season, period and age at first calving [AFC] groups) on 11 MTDFY records (from 6^th^, 36^th^, 66^th^, and 300^th^ day of lactation) and L305FY records. The data were classified into different seasons, periods and AFC groups. Each year was classified into four seasons on the basis of rainfall, temperature and humidity over the years-winter (December-March), summer (April-June), rainy (July-August), and autumn (September-November). The data spread over 22 years were classified into 10 periods. The data were classified into 9 AFC sub-groups using Sturges’ formula [8]. Fat percentage was determined by Lacto Star apparatus (German equipment produced by Funke-Gerber). For calibration of Lacto Star apparatus, fat percentage of milk was tested by Gerber method [9].

### Statistical methods

The least squares maximum likelihood program of Harvey [10] was used to estimate and study the effect of genetic and non-genetic factors on MTDFY and L305FY records of Murrah buffaloes:

Y_ijklmn_ = μ + PA_i_ + S_j_ + P_k_ + A_l_ + B_m_ + e_ijklmn_

Where, Y_ijklmn_ = Observation on the n^th^ individual in i^th^ parity, j^th^ season, k^th^ period, l^th^ AFC group and sired by m^th^ bull, μ = Overall population mean, PA_i_ = Effect of i^th^ parity (1-4), S_j_ = Effect of j^th^ season (1-4) four seasons: Winter (December to March), summer (April to June), rainy (July to August), autumn (September to November) were considered for analysis, P_k_ = Fixed effect of k^th^ period of study (1-10) a period in a block of 2 years was considered, A_l_ = Fixed effect of l^th^ AFC group, B_m_ = Random effect of m^th^ bull (sire), e_ijklmn_ = Random error, NID (0, σ^2^_e_).

### Estimation of heritability

Paternal half-sib correlation method given by Becker, 1975 [11] was used to estimate the heritability of different characters and their genetic correlations. A total of 75 bulls having three or more number of progeny were included for the estimation of heritability. The data were adjusted for those non-genetic factors showing significant effects and further used for estimation of heritability. The standard error of heritability was estimated as per Swiger *et al*. [12].

### Genetic and phenotypic correlations

The genetic and phenotypic correlations among MTDFY and 305-days fat yield were calculated from the analysis of variance and covariance among sire groups as given by Becker [11] and shown in below.

r_g(XY)_ = Cov S_xy_/√ σ^2^_s(x)_. σ^2^_s(y)_

## Results and Discussion

The least squares mean along with their standard errors for MTDFY and L305FY are shown in Table-1. The highest MTDFY was observed in MTDFY-3 (7.06 kg), and the lowest was observed in MTDFY-11 (3.33 kg). In general, MTDFY increased until MTDFY-3 and thereafter a gradual decline was noticed until the end of lactation. The overall mean of average 305-day fat yield was 175.74±4.12 kg. Ibrahim *et al*. [13] and Tonhati *et al*. [14] reported overall mean of 305-day fat yield as 147.67 kg and 90.1 kg in Egyptian buffaloes and Murrah buffaloes herd in Sao population, respectively, which was comparatively lower than Murrah breed in this study.

**Table-1:** Least squares means of L305FY and different MTDFY (in kg).

Trait	N	Mean±SE	CV (%)
MTDFY1	1049	6.81±0.184	39.44
MTDFY2	1049	7.05±0.18	33.41
MTDFY3	1049	7.06±0.17	31.72
MTDFY4	1049	6.55±0.14	31.34
MTDFY5	1048	6.11±0.13	33.12
MTDFY6	1028	5.56±0.16	34.33
MTDFY7	1002	5.15±0.17	36.80
MTDFY8	949	4.56±016	38.42
MTDFY9	869	4.73±0.13	42.76
MTDFY10	722	3.74±0.15	44.01
MTDFY11	567	3.33±0.14	48.09
L305FY	1049	175.74±4.12	26.55

N=Number of observation, L305FY=Lactation 305-fat yield, MTDFY=Monthly test day fat yields, SE=Standard error, CV=Coefficient of variation in percentage

### Effect of non-genetic factors

#### Parity

The effect of parity was highly significant (p<0.01) up to MTDFY-6 and L305FY; non-significant effect of parity was observed in rest TD (Table-2). Singh *et al*. [15] observed the non-significant effect of parity on fat percentage in Murrah buffaloes. Similar results were shown by Shah and Schermerhorn [16] in Nilli-Ravi buffaloes.

**Table-2:** Mixed model ANOVA showing mean sum of squares for factors affecting MTDFY and L305FY.

Traits	Sire	Parity	Season	Period	AFC group	Error
d. f.	74	3	3	9	8	951
MTDFY1	7.38*	154.86**	22.90**	11.56*	7.67^NS^	5.37
MTDFY2	6.88**	85.91**	30.84**	10.91*	9.97*	4.625
MTDFY3	6.19**	70.12**	40.11**	8.05*	11.69**	4.03
MTDFY4	4.80^NS^	35.96**	2.78^NS^	6.18^NS^	7.16^NS^	3.86
MTDFY5	4.51^NS^	28.21**	3.54^NS^	8.90*	9.19*	3.66
MTDFY6	5.91**	23.46**	6.47^NS^	6.85^NS^	7.99*	3.93
MTDFY7	6.46**	9.97^NS^	9.48^NS^	9.11*	4.47^NS^	4.36
MTDFY8	5.83*	3.60^NS^	15.24*	7.97^NS^	4.51^NS^	4.35
MTDFY9	4.93^NS^	7.99^NS^	19.435**	10.58*	4.87^NS^	4.66
MTDFY10	5.40^NS^	3.06^NS^	22.53**	11.75**	3.06^NS^	4.58
MTDFY11	4.67^NS^	2.98^NS^	21.20**	11.68**	2.55^NS^	3.89
L305FY	3521.85**	44925.29**	16778.69**	11805.05**	1353.61^NS^	2301.15

* p≤0.05,

** p≤0.01.

NS=Non-significant, L305FY=Lactation 305-fat yield, MTDFY=Monthly test day fat yields, AFC=Age at first calving

#### Season

The effect of season of calving was highly significant (p<0.01) up to MTDFY-3 then MTDFY-9 to MTDFY-11 and L305FY; significant (p<0.05) for MTDFY-8. Non-significant effect of season of calving was observed MTDFY-4 to MTDFY-7 (Table-2). Ibrahim *et al*. [13] and Mourad *et al*. [17] reported significant effect of season of calving on lactation fat yield in Egyptian buffaloes. Khan *et al*. [18] also reported a significant effect of season of calving on fat yield in Nilli Ravi buffaloes. In Murrah buffaloes, Hatwar [19] found significant effect of season of calving on fat yield and fat percentages.

#### Period

Highly significant effect (p<0.01) of the period of calving was observed for L305FY and MTDFY-10 and MTDFY-11; significant (p<0.05) for MTDFY-1, MTDFY-2, MTDFY-3, MTDFY-5, MTDFY-7, and MTDFY-9. Non-significant effect of period of calving was observed MTDFY-4, MTDFY-6, and TDFY-8 (Table-2). Ibrahim *et al*. [13] and Mourad *et al*. [17] observed significant effect of period of calving on lactation fat yield in Egyptian buffaloes. Khan *et al*. [18] also found a significant effect of period of calving of fat yield in Nili Ravi buffaloes.

#### AFC groups

The effect of AFC groups on MTDFY is presented in Table-2. A significant effect (p<0.01) of the AFC was observed on MTDFY-3; significant (p<0.05) for MTDFY-2, MTDFY-5, MTDFY-6 and rest TD have non-significant effect of AFC groups. Non-significant effect of AFC groups was also observed on L305FY. Shah and Schermerhorn [16] reported non-significant effect of AFC on fat percentage in Nilli-Ravi buffaloes.

### Genetic and phenotypic parameters

#### Heritability

The heritability of the MTDFY is shown in Table-3. The h^2^ estimate of MTDFY was the lowest (0.06) for MTDFY-8 and the highest (0.43) for MTDFY-3 and L305FY heritability was 0.33. Madad *et al*. [20] observed that heritability estimates ranged from 0.03 to 0.24 for TD fat percentages in Iranian buffaloes. Ibrahim *et al*. [13] reported heritability of lactation fat yield in Egyptian buffaloes as 0.19. Aspilcueta-Borquis *et al*. [21] found heritability estimate as 0.23 for L305FY in buffaloes. In Murrah buffaloes, Tonhati *et al*. [14] observed heritability estimate of lactation fat yield as 0.21.

**Table-3:** Heritability estimates along with their standard error for L305FY and different MTDFY.

Trait	N	h^2^±SE	Trait	N	h^2^±SE
MTDFY1	1049	0.28±0.16	MTDFY7	1002	0.29±0.16
MTDFY2	1049	0.37±0.17	MTDFY8	949	0.06±0.14
MTDFY3	1049	0.43±0.18	MTDFY9	869	0.22±0.12
MTDFY4	1049	0.30±0.17	MTDFY10	722	0.18±0.15
MTDFY5	1048	0.41±0.18	MTDFY11	567	0.13±0.15
MTDFY6	1028	0.40±0.18	L305FY	1049	0.33±0.16

N=Number of observation, L305FY=Lactation 305-fat yield, MTDFY=Monthly test day fat yields, SE=Standard error

#### Genetic and phenotypic correlations

The estimates of genetic and phenotypic correlations among 305-day fat yield and MTDFY are shown in Table-4. The estimates of genetic and phenotypic correlations between 305-day fat yield and MTDFY ranged from 0.51 to 0.99 and 0.32 to 0.48, respectively. Estimate of genotypic and phenotypic correlation between traits was similar to Sahoo *et al.*, 2014 [22]. MTDFY-5 had the highest genetic (0.99) and phenotypic (0.48) correlation with L305FY. Records up to five months can provide the similar results to lactation fat yield with almost 99% accuracy. Therefore, instead of 11 months with L305FY analysis can be made based on MTDFY-5.

**Table-4:** Genetic and phenotypic correlations among L305FY and different MTDFY.

Trait 1	Trait 2	Genetic correlations	Phenotypic correlations
L305FY	MTDFY1	0.87±0.28	0.32
	MTDFY2	0.63±0.26	0.39
	MTDFY3	0.92±0.20	0.42
	MTDFY4	0.90±0.30	0.45
	MTDFY5	0.99±0.30	0.48
	MTDFY6	0.99±0.18	0.46
	MTDFY7	0.99±0.19	0.47
	MTDFY8	0.88±0.24	0.46
	MTDFY9	0.99±0.19	0.46
	MTDFY10	0.51±0.41	0.45
	MTDFY11	0.73±0.36	0.43

L305FY=Lactation 305-fat yield, MTDFY=Monthly test day fat yields

## Conclusions

In this study, all the genetic and non-genetic factors except AFC group significantly affected the considered traits. The h^2^ estimate of lactation milk fat yield was around 0.33 and it ranged from 0.06 (MTDFY-8) to 0.43 (MTDFY-3). The estimates of phenotypic and genetic correlations of monthly TD yields with 305-day fat yield were generally higher in MTDFY-5 of lactation suggesting that this 5^th^ TD fat yields could be used as the selection criteria for early evaluation and selection of Murrah buffaloes.

## Authors’ Contributions

Research work was done by MK. The experiment was designed and supervised by VV. PR, JV and CSP assisted MK in data recording, literature collection and data analysis, respectively. AKC provided valuable suggestion regarding design of experiment and data analysis. VV and MK compiled the results as well as the manuscript. All authors read and approved the final manuscript.
